# Radiographic Evaluation of Maxillary Anterior Teeth Canal Curvatures in an Iranian Population

**Published:** 2011-02-15

**Authors:** Farida Abesi, Maryam Ehsani

**Affiliations:** 1. Department of Oral and Maxillofacial Radiology, Dental school, Babol University of Medical Sciences, Babol, Iran.; 2. Department of Endodontics. Dental school, Babol University of Medical Sciences, Babol, Iran.

**Keywords:** Maxillary Anterior Teeth, Radius of Curvature, Root Curvature

## Abstract

**INTRODUCTION:**

Complete knowledge of root canal curvature is a critical factor in successful endodontic treatment. The aim of this study was to investigate the direction, radius and degree of curvature of maxillary anterior teeth and the relationship between the radius and degree of curvature in Babol, a northern city of Iran.

**MATERIALS AND METHODS:**

A total of 242 radiographs of maxillary anterior teeth (central, lateral and canine) were taken by periapical parallel technique and processed by automatic processing. The degree of canal curvature was measured only at mesiodistal direction with Schneider method and classified according to Seidberg method. Statistical analysis was performed with Kruskal Wallis and Mann-Whitney U tests.

**RESULTS:**

Overall, 153 (62%) teeth had curvatures; 35.3% were mesially inclined and 64.7% were distally inclined. The degree of canal curvature was categorized into small, intermediate, and severe, that is 39.3%, 44.6% and 16.1%, respectively. The mean value of root curvature angle was 7.24°±9.03° in central incisors, 12.08°±11.02° in lateral incisors, and 15.08°±12.02° in canines respectively. There was significant correlation between type of tooth and degree of curvature (P=0.000). Significant correlation was not found between the type of tooth and radius of curvature (P=0.365).

**CONCLUSION:**

In the present study, 62% of maxillary anterior teeth had some form of curvatures; highest degrees of curvature were attributed to the canine teeth.

## INTRODUCTION

The main object of root canal therapy is the elimination of microorganisms and infected tissue from the tooth root canal system. This is performed by enlarging and shaping the canals to allow for adequate chemical debridement, while at the same time preserving the original shape and structure of the tooth [1]. Thorough knowledge of the anatomical configurations of the dental pulp, and the possible variations is critical for successful endodontics [2]. Conducting root canal therapy (RCT) when uncertain of the canal morphology increases the risk of transportation, ledge formation and even perforation, and often results in failure of the root canal procedure [3][4][5]. Several techniques are used to determine root canal configuration such as specimen transparent technique, conventional radiographs, radiopaque contrast media, cross-sectional cutting scanning electron microscopy (SEM) and cone beam computed tomography (CBCT) [6][7][8][9][10][11].

Schneider divided the root canal curvature into different root angles [12]. However, evaluating the curvature by the angle of the canal is now thought to be insufficient. For instance, two canals with the same angle obtained by the Schneider method could have very different radii of curvature or abruptness of curvatures [13]. Pruett et al. proposed that the assessment of canal curvature should be indicated by two measurements: a) the angle of curvature and b) the radius of curvature determined mathematically from radiographs [14]. There is a general shortage of clinical studies on canal curvature of maxillary anteriors as most investigations have concentrated on the type and variation of the root canal system [8][15]. Few investigations have been conducted on the specific degree of canals curvature of the maxillary anterior teeth [16]. The purpose of this study was to determine the degree, direction and radius of canal curvature in maxillary anterior teeth in Babol, a northern city of Iran.

## MATERIALS AND METHODS

This study was performed on 242 radiographs of anterior teeth (central, lateral, and canine) taken from patients referred to a maxillofacial radiology clinic in Babol, Iran. The patients were initially informed about the study and upon agreement they filled the inform consent form. Teeth that had not received endodontic therapy and had complete apical foramen and contour were selected. All radiographs were taken by periapical parallel technique with film holder XCP (Dentsply, United Kingdom) and processed by automatic processor (Velopex, United Kingdom).

**Figure 1:**
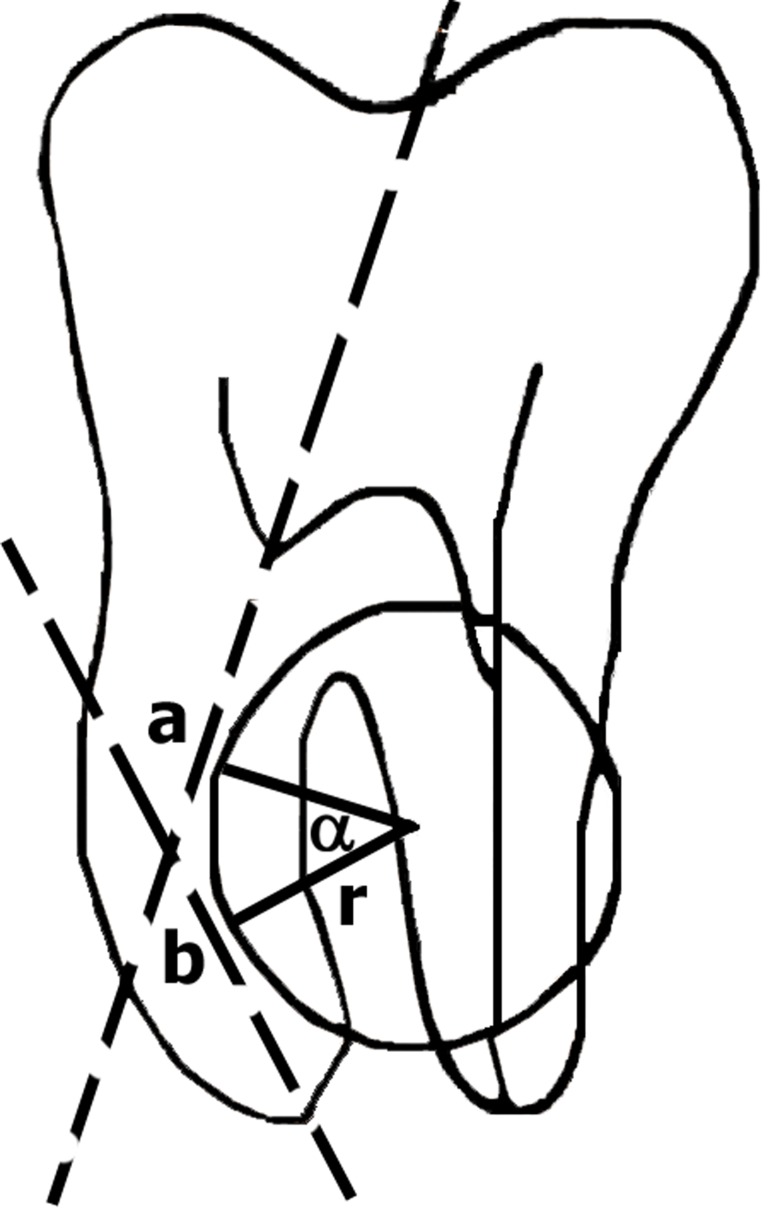
Angel (α) and Radius (r) of root curvature

The angle of curvature was determined by the Schneider technique which measures curvature as well as the acute angle between the long axis of the canal and a line joining the apical foramen to the point of initial curvature shown on the radiograph (Figure 1) [12]. Teeth were classified according to Seidberg classification; the degree of the curvature was categorized as low (<5°), moderate (5-25°) and severe (25-70°). The radius of curvature was measured by the Estrella method; that is, two 6-mm semi straight lines superimposed to root canal (Figure 1) [11]. According to this method, the first line (line b) represents longer continuity of the apical region and the second line (line a) represents the middle and cervical thirds. Of the second line, the nearest 6mm to the first line was considered; the lines perpendiculars to the midpoints of two 6-mm semi straight lines convene each other at the point that is Circum Center. This point is the center of the circle which determines the magnitude of the root curve [11]. The curvature measurement is performed by two observers with conveyor (Rotting, Germany) with accuracy of one degree. According the Estrela, the root curvature radius was classified as following:

-Small radius (≤4mm) i.e. severe curvature

-Intermediate radius (4<r≤8) i.e. moderate curvature

-Large radius (r>8mm) i.e. mild curvature

The data were analyzed with Kruskal Wallis and Mann-Whitney U tests. For multiple comparisons bonferroni adjustment of α was carried out.

## RESULTS

Out of 242 examined teeth, 153 (62%) had curvatures; 35.3% were mesially and 64.7% were distally inclined (Table 1).

**Table 1:** Frequency of mesial+distal curvatures among the teeth

**Direction**	**Mesial**	**Distal**	**Total**
**Frequency (%)**	54 (35.3)	99 (64.7)	153 (100.0)

The degree of canal curvature was as follows: 39.3% with small curvature, 44.6% with intermediate and 16.1% with sever curvature (Table 2).

**Table 2:** Frequency and percentage of angles of curvatures among the teeth

**Angle**	**≤4**	**5-24**	**≥25**	**Total**	
**Frequency (%)**	95(39.3)	108 (44.6)	39 (16.1)		242 (100.0)

Distribution of different curvature radius (according to Estrela) in studied teeth is presented in Table 3.

**Table 3:** Frequency and percentage of radius in teeth with angles over 20

**Radius**	** ≤4**	** 5-8**	**≥9**	**Total**
**Frequency (%)**	2 (2.5)	15 (19.0)	62 (78.5)	79 (100.0)

The tooth type showed no significant correlation with radius of curvature (P=0.365) (Table 4), and significant correlation with curvature angle (P=0.000) (Table 5). Mann-Whitney U test shown significant difference between central and lateral (P=0.003), central and canine (P=0.000) but the difference between lateral and canine was not significant (P=0.128).

**Table 4:** Mean and standard deviation of radius according to tooth type

**Tooth**	**Number**	**Mean±SD**	**P value**
**Central**	18	12.44±3.36	0.365
**Lateral**	35	12.00±5.00	
**Canine**	26	10.84±3.22	

**Table 5:** Mean and standard deviation of degree of curvature according to tooth type

**Tooth**	**Number**	**Mean±SD**	**P value**
**Central**	86	7.24±9.03	0.000
**Lateral**	100	12.08±11.02	
**Canine**	56	15.08±12.02	

## DISCUSSION

Radiographs are commonly utilized in endodontics to determine canal morphology; however the two dimensional image of the teeth might cause some uncertainty as to the actual direction and the degree of root canal curvature. Radiographs are essential to the practice of endodontics [17]; when assessing root canal curvatures parallel technique is the method of choice as it provides a less distorted view of the dentition, as well as clearer apical areas. Bisecting angle technique may be used if there are anatomic constraints such as a shallow palate. However, in this study these cases were excluded. Endodontic cases were also excluded, as K-files do not exactly conform to the canal shape, and will not always remain centered in the canal lumen, moreover iatrogenic errors may occur [18][19]. Several studies [14][20][21][22][23][24][25][26] have suggested methods to determine root canal curvature using periapical radiographs. Our technique determined angle of root curvature based on Schneider method [12].

In present study, the degree of root canal curvature of maxillary canines was larger than that of maxillary incisors concurring with the findings of Tao et al. They investigated root canal curvature in 400 maxillary anterior teeth by indirect digital radiography from both labiolingual and mesiodistal direction. He also concluded that the radius of curvature of maxillary canines root canals were smaller than that of maxillary incisors [16]. However, our study was not able to find a significant relation between type of tooth and the radius curvature (P=0.365).

Estrella et al. described a new method to determine root curvature radius in CBCT images [12]. According to this method we measured the radius of root curvature and found that there was inverted relation between radius and degree of root curvature; however this was not significant (r=0.155, P>0.05).

Our results agree with two previous studies that found no significant difference between the degree and the radius of curvature of root canals; also no relationship existed between the original radius of curvature and apical transportation [14][20]. However, determination of root canal curvature by use of its radius has proven to be an effective method.

Further studies should concentrate on CBCT images of teeth in buccolingual and mesiodistal directions, keeping dose limitation in mind.

## CONCLUSION

In the present study, 62% of maxillary anterior teeth had root curvatures and the lowest degree of curvature was related to the centrals. The results of this study can enhance endodontic therapy predictability and minimize errors during post insertion.
